# Passive virus movements with organelle dynamics

**DOI:** 10.18632/oncotarget.5897

**Published:** 2015-09-28

**Authors:** Kazuya Ishikawa, Masayoshi Hashimoto, Shigetou Namba

**Affiliations:** Laboratory of Plant Pathology, Department of Agricultural and Environmental Biology, Graduate School of Agricultural and Life Sciences, The University of Tokyo, Tokyo, Japan

**Keywords:** plant virus, intracellular trafficking, endoplasmic reticulum, actin filaments, particle formation

Eukaryotic cells are characterized by their dynamics. Intracellular dynamics are mainly generated as a function of the cytoskeleton, which plays essential roles in a diverse array of cellular processes: transport of macromolecules and endomembranes, spatial arrangement of organelles, and maintenance and alteration of cell morphology [1]. The motive force for trafficking along the actin cytoskeleton is provided by actin filaments and myosin motors [2]. All myosin molecules have an N-terminal motor domain and a C-terminal cargo-binding domain. Plant myosins consist of two plant-specific subfamilies, class VIII and class XI myosins; the latter are major contributors to organelle dynamics.

The plant endoplasmic reticulum (ER) is a highly dynamic organelle that continuously streams throughout the cytoplasm. The ER, running in parallel with the actin filaments, forms an actin-ER network. Actin filaments and XI-1, XI-2 and XI-K myosins mainly provide the motive force of the ER, whose stream probably facilitates the diffusion of molecules throughout the cytoplasm [3]. Simultaneously, the stream of the ER is proposed to drag the surrounding cytosol, which causes cytoplasmic streaming [3].

Many plant viruses have proteins that form cytoplasmic inclusions when expressed in plant cells, and some of these inclusions move rapidly along the actin-ER network [4]. Such intracellular movements of the viral protein inclusions are generally believed to be caused by direct recruitment of the myosin motors for their cell-to-cell movement. However, the mechanism and biological significance of the motility remain unclear.

**Figure 1:**
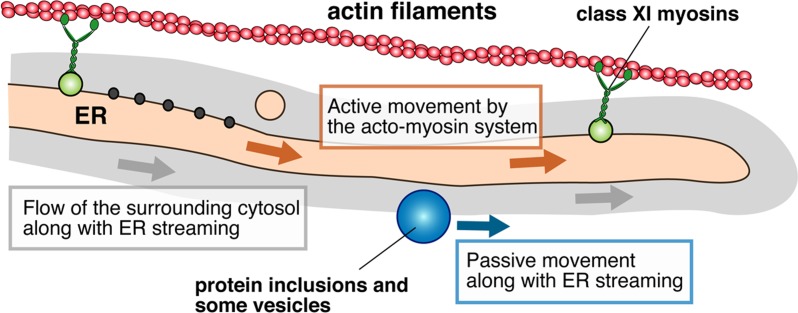
A model of the passive movement along with ER streaming

We studied intracellular trafficking of a plant RNA virus, fig mosaic virus (FMV), which has enveloped particles similar to animal viruses [5]. Using confocal scanning laser microscopy (CSLM), we found that the FMV nucleocapsid protein (NP), a major structural protein, forms cytoplasmic agglomerates (hereafter called NP bodies or NBs) that rapidly move along the actin-ER network when ectopically expressed in plant cells. Ultrastructural analysis of the NP-expressing cells using immunogold labeling and electron microscopy revealed that NBs localized in the cytosol in close proximity to the ER. Treatment with latrunculin B (LatB), an actin polymerization inhibitor, halted the movements of NBs, suggesting that the acto-myosin system provides the motive force for NB movement. To assess the mechanism of NB motility in more detail, we employed a dominant-negative form of class XI myosins, XI-1, XI-2, and XI-K, which lack the N-terminal motor domain. In accordance with the previous report that XI-1, XI-2, and XI-K play a pivotal role in ER motility [6], these dominant-negative myosins inhibited the stream of the ER. Likewise, NB movement was affected by the dominant-negative myosins in a similar pattern. However, CSLM observation and immunoprecipitation assays did not suggest either co-localization or interaction between NP and the dominant-negative myosins, indicating that NBs were indirectly moved by XI-1, XI-2, and XI-K myosins. The localization of NP in FMV-infected cells was also investigated using immunogold labeling and electron microscopy. As was the case when NP was ectopically expressed, NP formed NBs and localized in the cytosol in close proximity to the ER. Moreover, some of the NBs were surrounded by the ER membrane, which appeared to be forming particles. These results suggest that NBs localize in the cytosol in close proximity to the ER to form the basis of enveloped virus particles, and are consequently caught in the streaming of ER and the surrounding cytosol (Figure 1).

Numerous studies have demonstrated intracellular movements of viral protein inclusions along the actin-ER network, which are frequently interpreted as the first step of cell-to-cell movement [4]. Viruses must pass through plasmodesmata (PD), channels providing the symplastic continuity between plant cells, for their cell-to-cell movements. In many cases, however, including our study, these inclusions are larger than PD, whose diameters are a few ten nanometers, whereas the diameters of the viral protein inclusions are a few hundred nanometers or more. Taken together with our results, it is unlikely that the movements of the viral inclusions along the actin-ER network are always directly involved in the cell-to-cell movements. Whenever the viral inclusions access the ER (for example for replication or particle formation), they can potentially show passive movements along the actin-ER network, as is the case with FMV.

Such passive movements may not be unique to plant viruses. A similar hypothesis has been proposed for endomembrane trafficking in plant cells [7]. In this hypothesis, some of the organelles and vesicles are forced to move by other organelles and vesicles, which traffic by using the acto-myosin system directly. In conclusion, intracellular dynamics must be considered for proper interpretation of live-cell images and for advancing our understanding of cell biology.
